# Abnormal Global Functional Connectivity Patterns in Medication-Free Major Depressive Disorder

**DOI:** 10.3389/fnins.2018.00692

**Published:** 2018-10-09

**Authors:** Lu Zhang, Huawang Wu, Jinping Xu, Junjie Shang

**Affiliations:** ^1^Lab of Learning Sciences, Graduate School of Education, Peking University, Beijing, China; ^2^The Affiliated Brain Hospital of Guangzhou Medical University (Guangzhou Hui’ai Hospital), Guangzhou, China; ^3^Institute of Biomedical and Health Engineering, Shenzhen Institutes of Advanced Technology, Chinese Academy of Sciences, Shenzhen, China

**Keywords:** major depressive disorder, fMRI, resting-state, global brain connectivity, functional connectivity

## Abstract

Mounting studies have applied resting-state functional magnetic resonance imaging (rs-fMRI) to study major depressive disorder (MDD) and have identified abnormal functional activities. However, how the global functional connectivity patterns change in MDD is still unknown. Using rs-fMRI, we investigated the alterations of global resting-state functional connectivity (RSFC) patterns in MDD using weighted global brain connectivity (wGBC) method. First, a whole brain voxel-wise wGBC map was calculated for 23 MDD patients and 34 healthy controls. Two-sample *t*-tests were applied to compare the wGBC and RSFC maps and the significant level was set at *p* < 0.05, cluster-level correction with voxel-level *p* < 0.001. MDD patients showed significantly decreased wGBC in left temporal pole (TP) and increased wGBC in right parahippocampus (PHC). Subsequent RSFC analyses showed decreased functional interaction between TP and right posterior superior temporal cortex and increased functional interaction between PHC and right inferior frontal gyrus in MDD patients. These results revealed the abnormal global FC patterns and its corresponding disrupted functional connectivity in MDD. Our findings present new evidence for the functional interruption in MDD.

## Introduction

Major depressive disorder (MDD) is a highly prevalent and worldwide psychiatric disorder causing severe societal and familial burdens (Mathers and Loncar, 2006). Brain structural changes, including gray matter volume of insula, amygdala, hippocampus, frontal and temporal cortex (Bora et al., 2012; Wang et al., 2017b), and surface morphological properties of hippocampus and amygdala have been widely reported in MDD patients (Chen et al., 2016). In addition, altered structural covariance between angular gyrus and amygdala, posterior cingulate cortex in MDD is also observed (Chen et al., 2017; Wu et al., 2017). Using resting-state functional magnetic resonance imaging (rs-fMRI), abnormal local brain activities in precuneus, cerebellum, lingual gyrus and inter-regional functional connectivity between subgenal anterior cingulate cortex and temporal cortex, between insula and thalamus, inferior parietal cortex, and between intraparietal sulcus and superior temporal gyrus (STG) were also identified (Wu et al., 2016a; Wang et al., 2017a,c; Sun et al., 2018; Wang J. et al., 2018). Moreover, using graph-theory method, disrupted whole brain functional topological organization of network has also been found (Gong and He, 2015). All these studies suggested that structure and function have changed in MDD. However, how and where the global functional connectivity patterns changed in MDD remains unclear.

A large number of literatures have revealed that brain function was constrained by its connectivity patterns (Passingham et al., 2002; Fan et al., 2014, 2016; Zhang et al., 2014; Wu et al., 2016b; Yang et al., 2016). A recently developed weighted global brain connectivity (wGCB) method can search for the global functional connectivity patterns based on resting-state functional connectivity (RSFC) MRI (Cole et al., 2010). Resting-state fMRI is a non-invasive way to study the functional interactions between different brain areas (Biswal et al., 1995; Wang et al., 2012, 2015a; Xu et al., 2015; Zhang et al., 2016; Wang L. et al., 2018). Resting-state fMRI has been widely adopted to characterize functional connectivity patterns to identify intrinsic functional modules (Fox et al., 2006; Buckner et al., 2009; Power et al., 2011; Yeo et al., 2011; Cole et al., 2014; Wang et al., 2015b, 2016b, 2017d; Mears and Pollard, 2016). It has also been applied to explore the abnormal functional couplings between brain areas to delineate brain intrinsic functional changes in disorders (Wang et al., 2016a; Wu et al., 2016c; Liu et al., 2018). wGCB can reveal global changes in the connectivity of a brain region by searching for globally connected or disconnected brain regions using a data-driven manner (Cole et al., 2010). Unlike the traditional seed-based or independent component analysis methods which can merely identify same spatial patterns of connectivity across subjects, the wGBC is less likely to be affected by within-region and between-subject spatial variations in connectivity patterns (Cole et al., 2011). Moreover, compared to unweighted GBC, wGBC does not need to threshold the connectivity strengths and can reveal globally connected regions with many low-strength connections removed by unweighted GBC thresholding. Therefore, wGCB provides a new way to study altered global functional connectivities to identify pathophysiology of MDD.

In this study, using resting-state fMRI, we examined the potentially abnormal global brain connectivity patterns and corresponding functional connectivity changes in 23 MDD patients and 34 gender-, age-, and education level-matched healthy controls (HC). First, we computed a voxel-wise wGCB maps for both MDD and HC to identify the abnormal global functional connectivities in MDD. Subsequently, we calculated the RSFC of the brain regions with changed wGCB to further reveal altered functional interactions in MDD.

## Materials and Methods

### Subjects

Twenty-three right-handed medication-free MDD patients and 34 right-handed HC subjects were recruited at the Department of Psychiatry at the Affiliated Brain Hospital of Guangzhou Medical University. The detailed information for MDD and HC subjects can be found in **Table 1**. MDD diagnosis was performed based on the Structured Clinical Interview of DSM-IV (SCID) criteria with 24-item Hamilton Depression Rating Scale. The HC subjects were recruited with SCID Non-Patient Edition. All the included MDD and HC subjects were out of serious medical, surgical illness, history of seizures, substance abuse, head trauma, and contraindications for MRI. All the subjects signed the written informed consent. All the experiments were approved by the ethics committees of the Affiliated Brain Hospital of Guangzhou Medical University.

**Table 1:** Demographics and clinical characteristics of the subjects used in present study.

Subjects	MDD	HC	*p-*value
Number of subjects	23	34	
Gender (male: female)	9/14	15/19	0.71
Age (mean ± SD)	30.48 ± 7.13	29.71 ± 7.09	0.69
Years of education (mean ± SD)	13.35 ± 3.89	14.18 ± 2.17	0.31
HDRS scores (mean ± SD)	34.30 ± 7.58		
Age of onset (years)	27 ± 7.44		
Duration of illness(months)	43.04 ± 58.18		
Episodes (*n*, patients)			
First	17		
Recurrence	6		
Family history of MDD (*n*, patients)	5		

*A Pearson chi-squared test was used for gender comparison. Two-sample *t*-tests were used for age, education comparisons. HDRS, Hamilton Depression Rating Scale scores; MDD, major depression disorder; HC, healthy control.*

### Resting-State fMRI Data Acquisition

Resting-state fMRI data were acquired using an eight-channel 3.0-Tesla Philips MR scanner (Achieva X-series, the Netherlands) in the Department of Radiology, the Affiliated Brain Hospital of Guangzhou Medical University, China. The foam padding and earplugs were used to reduce head motion and to muffle scanner noise, respectively. During scanning, all the subjects were asked to stay awake, close their eyes, and think nothing. Resting-state fMRI data were scanned using an echo planar imaging sequence with the following parameters: repetition time = 2000 ms, echo time = 30 ms, flip angle = 90^0^, field of view = 220 × 220 mm^2^, matrix = 64 × 64, 33 slices, slice thickness = 4 mm with 0.6 mm gap, and 240 volumes. The resting-state fMRI data have been used in our previous studies (Wu et al., 2016a; Wang et al., 2017a).

### Resting-State fMRI Data Pre-processing

The resting-state data were pre-processed using SPM8 toolkit^1^. The pre-processing includes the following steps. discarding the first 10 volumes; slice timing; head motion correction; normalizing to MNI space; regressing motion parameters, white matter, cerebrospinal fluid, and global signals; and filtering with a temporal band-path of 0.01–0.1 Hz. To exclude the head motion effects, resting-state fMRI images with head-movement exceeded 1.5 mm of translation or 1.5 degrees of rotation in any direction were discarded if. In addition, “Scrubbing” method was also used to further reduce the effects of head motion if the frame displacement (FD) > 0.5 (Power et al., 2012). In our study, no frame was deleted because all subjects’ FD values were smaller than 0.3. For RSFC analyses, the resting-state data were first smoothed (6 mm FWHM) after normalization and then for the following pre-processing. We did not regress out the global signal to obtain reliable results because global signal regression will exaggerate anti-correlation.

### wGCB Analysis

The calculation of voxel-wise wGCB map was constrained by a gray matter mask with gray matter probability value > 0.2 (Wu et al., 2016a). The wGBC was calculated as the following steps. First, each voxel of the gray mask was selected as the seed voxel. Next, Pearson’s correlations coefficient was calculated between each seed voxel and each of the whole brain voxel and transformed to *z* value using Fisher’s *z* transformation. Then, all the correlations were averaged and transformed back to *r* value for this voxel presenting the average connectivity (Cole et al., 2010). Using the same procedure, a whole brain wGCB map was obtained for each subject and smoothed using a 6 mm FWHM Gaussian kernel before statistical analyses. The distributions of wGCB in HC and MDD subjects were identified using one-sample *t*-tests and the significant level was set at *p* < 0.05, cluster-level correction with voxel-level *p* < 0.001.

To determine the group differences in wGCB, the two-sample *t*-test was used to compare the wGCB maps between HC and MDD patients with age, gender, and education as covariates. The significant level was set at *p* < 0.05, cluster-level correction with voxel-level *p* < 0.001 after using a cluster-level Monte Carlo simulation with 5000 times.

### Functional Connectivity Analyses

Whole brain RSFC analyses were used to determine the changed functional connectivity of the brain regions with changed wGCB between MDD and HC. Functional connectivity was computed and transformed to *z* value using Fisher’s *z* transformation for each subject. A two-sample *t*-test was used to compare the functional connectivity maps between HC and MDD patients with age, gender, and education as covariates. The significant level was set at *p* < 0.05, cluster-level correction with voxel-level *p* < 0.001.

## Results

### Demographics and Clinical Characteristics

A chi-squared test and two-sample *t*-tests found that there were no significant differences in gender (*p* = 0.71), age (*p* = 0.69), and education level (*p* = 0.31) between HC and MDD groups.

### wGCB Distribution in MDD and HC

**FIGURE 1:**
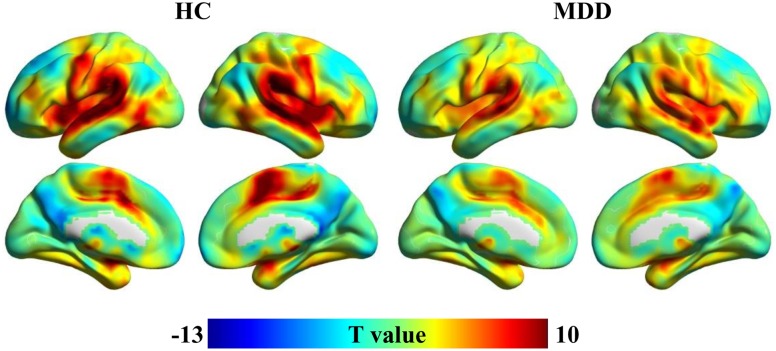
One-sample *t*-tests were used to identify the distribution of weighted global brain connectivity (wGCB) in major depressive disorder (MDD) and healthy controls (HC). The high wGCB were primarily detected in the superior temporal gyrus, lateral occipital gyrus, fusiform gyrus, intraparietal sulcus, medial temporal lobe, middle cingulate cortex, caudate, medial frontal cortex, lateral prefrontal cortex, inferior frontal cortex, and insula.

Spatial distribution patterns of wGCB in MDD and HC found that the high wGCB were primarily located in the STG, lateral occipital gyrus, fusiform gyrus, intraparietal sulcus, medial temporal lobe, middle cingulate cortex, caudate, medial frontal cortex, lateral prefrontal cortex, inferior frontal cortex, and insula (**Figure 1**).

### Changed wGCB in MDD

**FIGURE 2:**
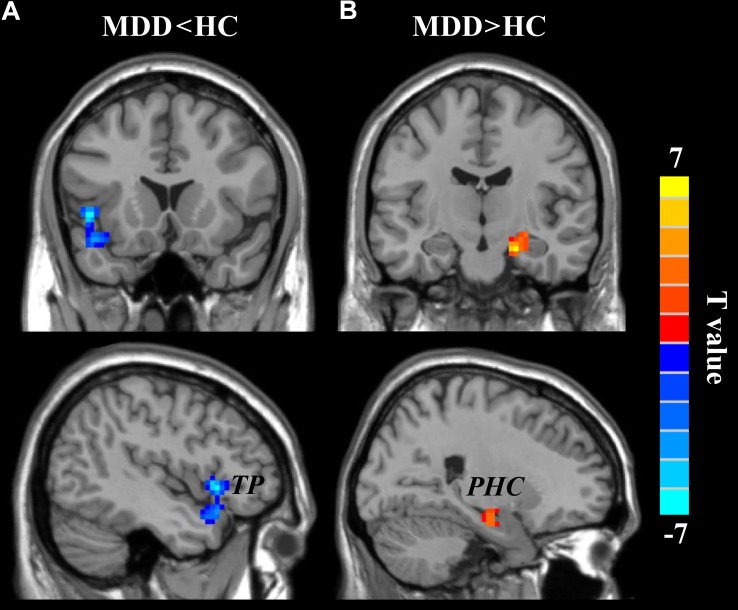
The changed weighted global brain connectivity (wGCB) in major depressive disorder (MDD) patients. Two-sample *t*-test was used to compare the wGCB maps between healthy controls (HC) and MDD patients and identified **(A)** decreased wGCB in left temporal pole (TP) and **(B)** increased wGCB in right parahippocampus (PHC). The significance was determined using a cluster-level Monte Carlo simulation (5000 times) corrected threshold of *p* < 0.05 (cluster-forming threshold at voxel-level *p* < 0.001).

A two-sample *t*-test (the significant level was set at *p* < 0.05, cluster-level correction with voxel-level *p* < 0.001) was applied to compare the wGBC and found significantly decreased wGCB in left temporal pole (TP) (peak MNI coordinate: [−48 18 −3], 91 voxels) and significantly increased wGBC in right parahippocampus (PHC) (peak MNI coordinate: [21 −12 −21], 55 voxels) in MDD patients (**Figure 2**).

### Altered Functional Connectivities

**FIGURE 3:**
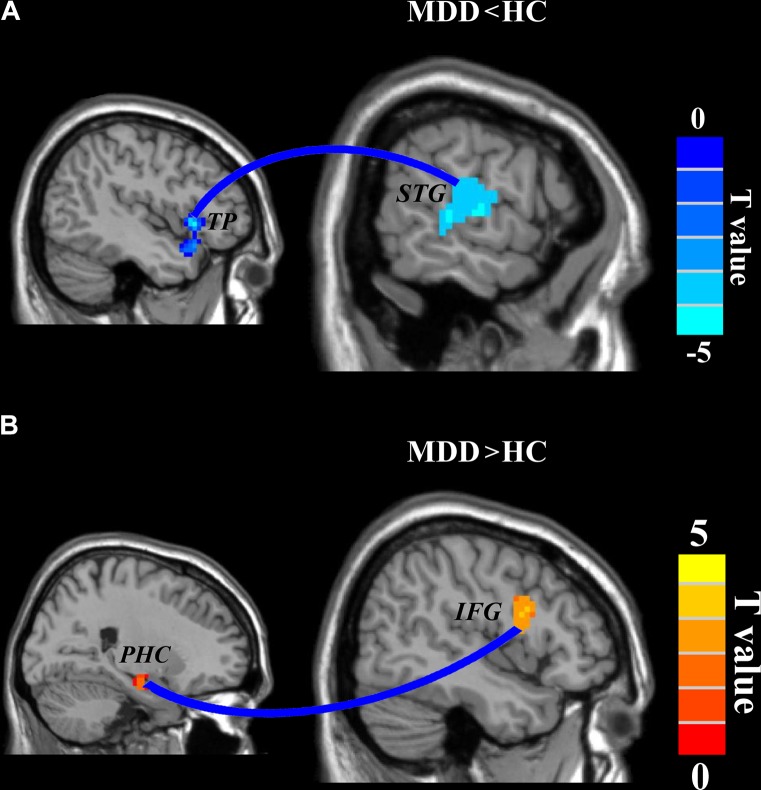
Disrupted functional connectivities in major depressive disorder (MDD) patients. Two-sample *t*-tests were used to identify the significant differences in functional connectivity between MDD and healthy control groups. **(A)** Significantly decreased functional connectivity between left temporal pole and right superior temporal gyrus and **(B)** significantly increased functional connectivity between right parahippocampus and right inferior frontal gyrus were found. The significance was determined using a cluster-level Monte Carlo simulation (5000 times) corrected threshold of *p* < 0.05 (cluster-forming threshold at voxel-level *p* < 0.001).

A two-sample *t*-test (the significant level was set at *p* < 0.05, cluster-level correction with voxel-level *p* < 0.001) was applied to compare the whole brain RSFC maps and identified significantly decreased functional connection between left TP and right posterior superior temporal gyrus (STG: peak MNI coordinate: [63 −15 3], 139 voxels) and significantly increased functional connection between right PHC and right inferior frontal gyrus (IFG: peak MNI coordinate: [42 3 24], 100 voxels) in MDD compared to HC (**Figure 3**).

## Discussion

In this study, we studied the changed global functional connectivity in MDD using wGCB method. wGCB analysis revealed decreased global functional connectivities in left TP and increased global functional connectivities in right PHC in MDD. The following functional connectivity analyses found decreased functional connectivity between left TP and right STG and increased functional connectivity between right PHC and right IFG in MDD. These findings suggested that abnormal emotion regulation and memory circuits play an important role in neuropathology of MDD.

Temporal pole and STG have been widely reported to be implicated in emotional processing and social cognition (Olson et al., 2007; Olson et al., 2013). TP is traditionally considered to participate in multimodal sensory integration (Skipper et al., 2011; Visser et al., 2012), but more and more studies have demonstrated that TP is also implicated in various high order cognitive functions, including face recognition (Olson et al., 2007), memory (Munoz-Lopez et al., 2010), and language processing (Hickok and Poeppel, 2007). The lateral TP which mainly connected with amygdala and orbital frontal cortex plays an important role in emotion regulation and theory of mind and is taken as a structure of emotional and social brain (Frith and Frith, 2010). The STG has also been reported to take part in emotional processing and social perception, especially the representation of emotional information during the initial stages of emotional regulation (Allison et al., 2000; Olsson and Ochsner, 2008). Structural and functional abnormalities of TP and STG in MDD were observed. Increased cortical thickness and decreased gray matter density of TP were identified in MDD (Fallucca et al., 2011; Peng et al., 2011; Igata et al., 2017). Abnormal functional activation of TP in MDD during sad emotion processing is also found (Beauregard et al., 2006; Keedwell et al., 2009). In STG, decreased gray matter volume and abnormal activity during sad response in MDD are also found (Fitzgerald et al., 2008; Takahashi et al., 2010). These studies indicated that TP and STG are two important nodes of affective network in emotion regulation. The decreased functional connectivity between TP and STG found in our study suggested that disconnectivity results in dysfunction of initial regulation of negative emotion in MDD patients.

The PHC which is an interface area between the hippocampus and the neocortex mainly takes part in memory function (Squire et al., 2004). PHC is also involved in recognition of emotional faces or scenes (Fitzgerald et al., 2008; Nummenmaa et al., 2008; Sabatinelli et al., 2011). The IFG plays a role in mood regulation (Baker et al., 1997; Northoff et al., 2000), associative emotional memory (Bookheimer, 2002; Price, 2003), and integrating emotional information and regulating the intensity of emotional responses (Cabeza and Nyberg, 2000; Fuster, 2001). PHC has been widely reported with decreased gray matter volume (Bora et al., 2012; Zhou et al., 2016), abnormal involvement during emotion and memory processing (Surguladze et al., 2005; Garrett et al., 2011; Palmer et al., 2014; Zamoscik et al., 2014), and damaged functional connectivity (Zeng et al., 2012). In our study, we found increased wGCB of PHC which is contrast to the gray matter volume changes. The inconsistency mainly results from gray matter volume and wGCB characterizing different properties of PHC. Structural and functional measurements of PHC may provide complementary evidence to better elucidate the role of PHC in MDD. During emotion processing in bipolar disorder during mania, hypoactivation of the IFG was observed during processing of negative faces (Altshuler et al., 2005), fear perception (Killgore et al., 2008), and negatively captioned pictures (Malhi et al., 2004). Functional disconnections of IFG have also been found in many previous studies (Murray et al., 2011; Tao et al., 2013). Moreover, reduced right IFG gray matter volume was found in MDD patients (Sabatinelli et al., 2011). All these studies suggested important roles of PHC and IFG in the pathology of MDD. In our study, we found increased functional connectivity between IFG and PHC in MDD patients compared to healthy controls. The hyperconnectivity between IFG and PHC indicated MDD patients need more efforts to inhibit negative emotion.

There are some limitations in this study. First, correlation analyses did not find significant associations between changed neuroimaging indices and HRSD scores. Thus, the conclusion needs to be further validated. Second, our samples are very small and these findings also need to be validated in a larger sample.

In conclusion, we used wGCB and functional connectivity analyses revealed abnormal global connectivity patterns in TP and PHC, and abnormal functional interactions between TP and STG, and between PHC and IFG. All these brain areas are parts of affective network and emotion regulation network. Our findings suggested that abnormal functional connectivity patterns of the two networks contribute to the pathology of MDD. The current findings will provide an important reference for future MDD therapy, including deep brain stimulation and transcranial magnetic stimulation.

## Author Contributions

JX and JS designed this study and revised the manuscript. HW collected the data. LZ analyzed the data and wrote the manuscript. All the authors discussed the results.

## Conflict of Interest Statement

The authors declare that the research was conducted in the absence of any commercial or financial relationships that could be construed as a potential conflict of interest. The handling Editor declared a past co-authorship with several of the authors HW and JX.

## Footnotes

1 https://www.fil.ion.ucl.ac.uk/spm/
